# Editorial: Immune involvement in recurrent pregnancy loss

**DOI:** 10.3389/fimmu.2023.1330701

**Published:** 2023-11-07

**Authors:** Gendie E. Lash, Raj Raghupathy

**Affiliations:** ^1^ Division of Uterine Vascular Biology, Guangzhou Institute of Pediatrics, Guangzhou Women and Children’s Medical Center, Guangzhou Medical University, Guangdong Provincial Clinical Research Center for Child Health, Guangzhou, China; ^2^ Department of Microbiology, College of Medicine, Kuwait University, Kuwait City, Kuwait

**Keywords:** recurrent pregnancy loss (RPL), uterine natural killer (uNK) cells, immune tolerance, regulatory T (Treg) cell, decidual macrophages, autoantibodies

Recurrent pregnancy loss (RPL) is defined as two or more consecutive pregnancy losses before the 20^th^ week of gestation, occurring in 1%–3% of reproductive age women. RPL is a devastating condition, significantly negatively impacting the quality of life of affected couples. Unexplained RPL (URPL) is a heterogeneous condition affecting approximately 50% of RPL cases, with one contributing factor thought to be a disruption in maternal immune tolerance. Various immune effectors and molecules in the uterine microenvironment establish and maintain specific maternal tolerance toward the semi-allogeneic fetus during pregnancy. Immune cells including innate lymphoid cells (ILCs), myeloid cells, T cells and B cells have been found to contribute to maintaining this maternal immunological tolerance during pregnancy. ILCs have been found to be the most abundant immune cells in the pregnant uterus, with many studies describing the relationship between RPL and either T cells or natural killer (NK) cells in peripheral blood and the endometrium/decidua. Despite progress in uncovering the roles of uterine NK and regulatory T cells in pregnancy, the immune heterogeneity in patients with URPL remains inadequately understood.

In this Research Topic, we aimed to collect manuscripts that would contribute to our understanding of the disruption of maternal immune tolerance during pregnancy leading to URPL.

This Research Topic contains 9 manuscripts covering both original articles and reviews. The collected articles highlight the heterogeneity associated with URPL, and recurrent implantation failure (RIF) which shares some features with RPL, and the need to further stratify these women to allow for personalized treatment options. In addition, it highlights some of the novel approaches being taken to identify aetiologies associated with these conditions. Zhang et al. used a novel integrated bioinformatics approach combining both antiphospholipid syndrome and RIF datasets to determine commonly altered genes, all of which were associated with the immune system. Their report suggests the possible identification of four candidate genes that could be considered for the diagnosis of RIF with antiphospholipid syndrome. However, the use of bioinformatics in RPL research was questioned due to the large heterogeneity of the population, often small study sizes and challenges in a standard definition of RPL and relevant risk factors, taking lessons from cancer research as a ‘gold standard’ (Betti et al.).

Autoantibodies are a major risk factor for reproductive failure; not just antiphospholipid antibodies, but those against other targets as well. Nørgaard-Pedersen et al. described the potential risks associated with thyroid-peroxidase and anti-nuclear antibodies in addition to anticardiolipin antibodies, β2 glycoprotein antibodies and lupus anticoagulant in conjunction with HLA-DR typing. Three studies further showcased the dysregulation of different immune cells with the endometrium/decidua in women with RPL, uNK cells, low-density granulocytes and macrophages, indicating changes in cell surface receptors as well as numbers being associated with this condition (Ye et al.; Woon et al.; Sang et al.). However, association does not necessarily point to causality and thus we still do not fully understand their contribution to the aetiology of URPL, or how they may be targeted therapeutically. Review articles covered the roles of uNK cells and trophoblast (Wei et al.), macrophages (Zhao et al.) and matrix metalloproteinases (Jing et al.) in both the non-pregnant endometrium as well as the early pregnant decidua. Wei et al. discussed the contributions of uterine NK cells to the essential remodeling of the spiral artery, while Zhao et al. elaborated on possible connections between recurrent spontaneous miscarriage and the phenotypes and functions of decidual macrophages.

We hope this Research Topic will be useful for researchers and clinicians alike to help inform patients as well as further studies. Developing stratifying criteria for the different sub-types of URPL, as well as potential therapeutic options for each of those sub-types is paramount.

## Author contributions

GL: Writing – original draft. RR: Writing – review & editing.

